# Breast Cancer Type Classification Using Machine Learning

**DOI:** 10.3390/jpm11020061

**Published:** 2021-01-20

**Authors:** Jiande Wu, Chindo Hicks

**Affiliations:** Department of Genetics, School of Medicine, Louisiana State University Health Sciences Center, 533 Bolivar, New Orleans, LA 70112, USA; jwu2@lsuhsc.edu

**Keywords:** gene expression, breast cancer, classification, machine learning

## Abstract

Background: Breast cancer is a heterogeneous disease defined by molecular types and subtypes. Advances in genomic research have enabled use of precision medicine in clinical management of breast cancer. A critical unmet medical need is distinguishing triple negative breast cancer, the most aggressive and lethal form of breast cancer, from non-triple negative breast cancer. Here we propose use of a machine learning (ML) approach for classification of triple negative breast cancer and non-triple negative breast cancer patients using gene expression data. Methods: We performed analysis of RNA-Sequence data from 110 triple negative and 992 non-triple negative breast cancer tumor samples from The Cancer Genome Atlas to select the features (genes) used in the development and validation of the classification models. We evaluated four different classification models including Support Vector Machines, K-nearest neighbor, Naïve Bayes and Decision tree using features selected at different threshold levels to train the models for classifying the two types of breast cancer. For performance evaluation and validation, the proposed methods were applied to independent gene expression datasets. Results: Among the four ML algorithms evaluated, the Support Vector Machine algorithm was able to classify breast cancer more accurately into triple negative and non-triple negative breast cancer and had less misclassification errors than the other three algorithms evaluated. Conclusions: The prediction results show that ML algorithms are efficient and can be used for classification of breast cancer into triple negative and non-triple negative breast cancer types.

## 1. Introduction

Despite remarkable progress in screening and patient management, breast cancer (BC) remains the second most diagnosed and the second leading cause of cancer deaths among women in the United States [1,2]. According to the American Cancer Association, there were 268,600 women newly diagnosed with BC in 2019, of which 41,760 died from the disease [1,2]. BC is a highly heterogeneous disease encompassing multiple types and many subtypes [3,4]. The majority of BCs respond to endocrine and targeted therapies, and generally have good prognosis and survival rates [3,4]. However, a significant proportion of BC are triple negative breast cancers (TNBC) [4,5]. TNBC is a specific subtype of BC characterized by lack of expression of the three most targeted biomarkers in BC treatment: estrogen receptor (ER), progesterone receptor (PR), and human epidermal growth factor receptor (HER-2) [2,6]. It accounts for 15% to 20% of all BCs diagnosed annually [4]. TNBC tumors are characterized by a more aggressive clinical behavior, poor prognosis, higher recurrence rates and poor survival rates [7,8,9,10,11,12,13,14]. Currently, there are no Food and Drug Administration (FDA) approved targeted therapies for this dreadful disease. Cytotoxic chemotherapy remains the main effective therapeutic modality, although some patients develop resistance and many others who survive surfer many side effects [15]. The long-term side effects of chemotherapy are well-known and include infertility, osteopenia and osteoporosis, heart damage and in rare cases leukemia, as well as financial losses, all of which can severely impact the quality of life for the survivors [15]. Thus, there is an urgent need for the development of accurate algorithms for identifying and distinguishing truly TNBC tumors which could be prioritized for specialized treatment from non-TNBC tumors that can be safely treated using endocrine or targeted therapeutics.

Traditionally, classification of breast cancer patients into those with TNBC and non-TNB has been largely determined by immunohistochemical staining [16,17]. Discordance in assessment of tumor biomarkers by histopathological assays has been reported [16]. Recently, Viale et al. compared immunohistochemical (IHC) versus molecular subtyping using molecular BluePrint and MammaPrint in a population of patients enrolled in MINDACT [17]. These authors also compared outcome based on molecular subtyping (MS) versus surrogate pathological subtyping (PS) as defined by the 2013 St. Gallen guidelines [18]. They discovered and concluded that molecular classification can help to identify a larger group of patients with low risk of recurrence compared with the more contemporarily used classification methodology including high-quality assessed Ki67 [16,17]. Moreover, while traditional classification methods have been relatively effective, they lack the accuracy and specificity to identify those breast cancers that are truly TNBC from non-TNBC. Therefore, novel approaches are needed to address this critical unmet need.

BC screening in the United States has been routinely performed with mammography, digital breast tomosynthesis, ultrasound and magnetic resonance [19,20,21]. These breast imaging modalities for BC screening have resulted in a new and growing field of radiomics [19,20]. Radiomics analysis using contrast-enhanced spectral mammography images in BC diagnosis has revealed that textural features could provide complementary information about the characterization of breast lesions [20]. Radiomics has also been used in BC classification and prediction [21]. However, molecular classification of BC into TNBC and non-TNBC has received little attention. Given that TNBC tends to affect younger premenopausal women who are not recommended for screening using mammography, there is a need for the development of new classification algorithms.

Recently, the application of machine learning (ML) to molecular classification of tumors has come into sharper focus [22,23,24]. ML methods have been applied to breast cancer survival prediction [22], for diagnostic ultrasound of TNBC [23] and breast cancer outcome prediction with tumor tissue images [24]. However, to date, ML has not been applied to classification of patients with TNBC and non-TNBC using RNA-sequence (gene expression) data. The objective of this study was to investigate the potential for application of ML to classification of BC into TNBC and non-TNBC using RNA-Sequence data derived from the two patient populations. Our working hypothesis was that genomic alterations in patients diagnosed with TNBC tumors and non-TNBC tumors could lead to measurable changes enabling classification of the two patient groups. We addressed this hypothesis by evaluating the performance of four ML algorithms using publicly available data on TNBC and non-TNBC from The Cancer Genome Atlas (TCGA) [25].

## 2. Materials and Methods

**Figure 1:**
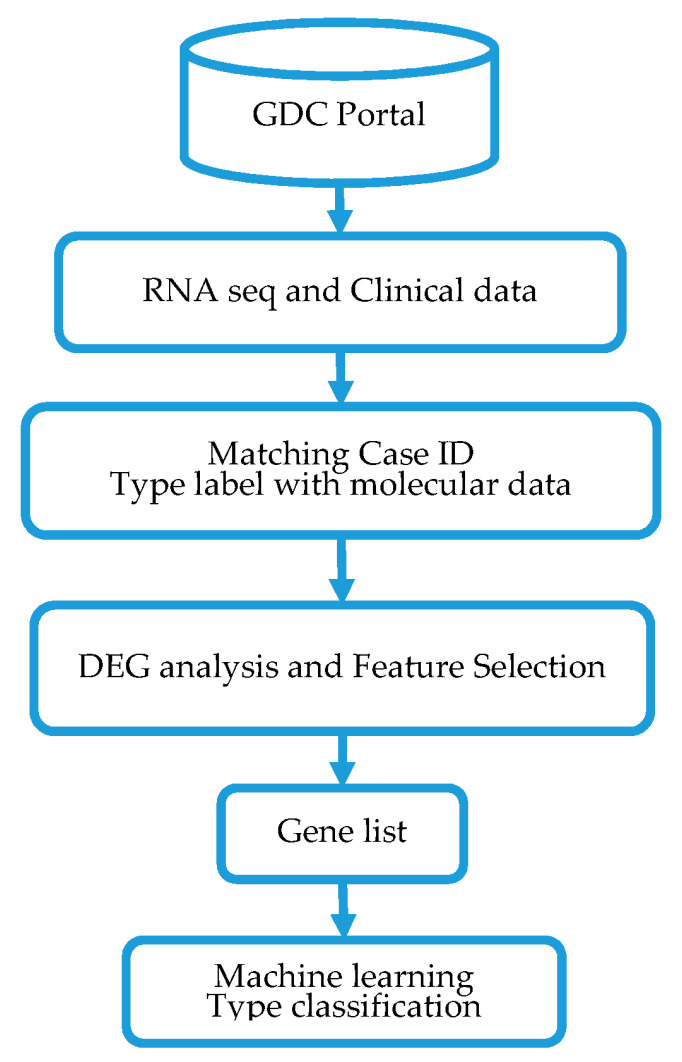
Project design, data processing and analysis workflow for classification of triple negative breast cancers (TNBC) and non-TNBC using machine learning method. GDC denotes the genomics data commons; DEG denotes differentially expressed genes.

The overall design and execution strategy used in this study is presented in Figure 1. Below we provide a detailed description of the sources of gene expression variation data along with clinical data used in this investigation, as well as the data processing and analysis strategies used.

### 2.1. Source of Gene Expression Data

We used publicly available RNA-Seq data on TNBC and non-TNBC from The Cancer Genome Atlas (TCGA) [25]. Gene expression data and clinical information were downloaded from the Genomics Data Commons (GDC) using the data transfer tool [26]. The data set included 1222 samples and 60,485 probes. Using the sample barcodes, we linked the gene expression data with molecular data and ascertained the samples as either TNBC or non-TNBC. Samples without clinical phenotyping or labels were excluded from the data sets and were not included in downstream analysis. We performed quality control (QC) and noise reduction on the original gene expression data matrix to remove rows with insufficient information or missing data. Due to the large difference in gene expression values, in order to facilitate later modeling and rapid training convergence, we normalized the expression profile data. The QCed data set was normalized using the LIMMA [27] and edgeR Bioconductor package implemented in R [27]. The probe IDs were matched with gene symbols using the Ensemble database. In our analyses, we used counts per million reads (CPM) and log-CPM. CPM and log-CPM values were calculated using a counts matrix alone and have been successfully used in RNA-Seq data processing [28]. After data processing and QC, the final data set used in downstream analysis consisted of 934 tumor samples distributed as 116 TNBC and 818 non-TNBC samples, and 57,179 probes. The probes were matched with gene symbols using the Ensemble database [29].

### 2.2. Differential Gene Expression Analysis and Feature Selection

The classification approach proposed in this article is a binary classification model. However, because of the large number of genes (herein called features) involved, which was much larger than the number of samples, the correlation between features was relatively complex, and the dependence between correlations was affected. This presented challenges in the application of ML. For example, with high dimensionality of the data, it takes a long time to analyze the data, train the model and identify the best classifiers. Therefore, as a first step, we addressed the data dimensionality problem to overcome the influence of unfavorable factors and improve the accuracy of feature selection. To address this need, we used various statistical methods.

Using a quality controlled normalized data set, we performed supervised analysis comparing gene expression levels between TNBC and non-TNBC samples to discover a set of significantly differentially expressed genes between TNBC and non-TNBC. For this differential expression analysis, we used the LIMMA package implemented in R [27]. We used the false discovery rate (FDR) procedure to correct for multiple hypothesis testing [30]. In addition, we calculated the log2 Fold Change (Log2 FC), defined as the median of gene expressed minus the gene expression value for each gene. Genes were ranked on FDR adjusted *p*-values and Log2 FC. Significantly (*p* < 0.05) differentially expressed genes were identified and selected. For feature selection, we used significantly differentially expressed genes between the two types of breast cancer as the features. These features were selected at different threshold levels.

### 2.3. Modeling Prediction and Performance Evaluation

As noted above, the research content of this paper was based on a binary classification model with application to pattern recognition classification problem [31]. Under this approach 90% of the data set was randomly selected as the training set and the remaining 10% as the test set. There are many methods for performing classification tasks [32], including Logistic Regression, Nearest Neighbor, Naïve Bayes, Support Vector Machine, Decision Tree Algorithm and Random Forests Classification [32]. In this investigation, we evaluated four methods for performance, including, Support Vector Machines (SVM), K-nearest neighbor (kNN), Naïve Bayes (NGB) and Decision tree (DT).

The basic model for Support Vector Machine is to find the best separation hyperplane in the feature space to maximize the interval between positive and negative samples on the training set. SVM is a supervised learning algorithm used to solve two classification problems. The K-nearest neighbor classification algorithm is a theoretically mature method and one of the simplest machine learning algorithms. The idea of this method is in the feature space, if most of the k nearest (i.e., the nearest neighbors in the feature space) samples near a given sample belong to a certain category, that sample also belongs to this category. Naïve Bayes is a generative model of supervised learning. It is simple to implement, has no iteration, and has high learning efficiency. It will perform well in a large sample size. However, because the assumption is too strong (assuming that the feature conditions are independent), it is not applicable in scenarios where the feature conditions of the input vector are related. Decision Tree is based on the known probability of occurrence of various situations by constructing a decision tree to obtain the probability that the expected value of the net present value is greater than or equal to zero, evaluate project risk, and determine its feasibility. DT is a graphical method of intuitive use for probability analysis.

The methods were evaluated for performance to identify the best performing algorithm, which was further evaluated. For each method, we repeated the modeling process 10 times and used a confusion matrix (CM) [33] to display the classification results. Due to the small data sets used, we performed a 10-fold cross-validation evaluation of the classification performance of the methods we tested to validate their performance. We also computed accuracy, sensitivity and specificity and used them as performance measures for comparing the four classification algorithms employed.

For evaluation and comparison of the classification and misclassification performance of the four ML algorithms, we used 4 different scenarios in which any sample could end up or fall into: (a) true positive (TP) which means the sample was predicted as TNBC and was the correct prediction; (b) true negative (TN) which means the sample was predicted as non-TNBC and this was the correct prediction; (c) false positive (FP) which means the sample was predicted as TNBC, but was non-TNBC, and (d) false negative (FN) which means the sample was predicted as non-TNBC, but was TNBC. Using this information, we evaluated the classification results of the model by calculating the overall accuracy, sensitivity, specificity, precision, and F1 Score indicators. These performance measures or indicators were defined and computed as follows:

Accuracy = (TP + TN)/(TP + FP + FN + TN). Recall = TP/(TP + FN) Specificity = TN/(TN + FP) Precision = TP/(TP + FP) F1 Score = 2 * (Recall * Precision)/(Recall + Precision)

To further validate the methods, the classification results were also compared with classic feature selection methods such as SVM-RFE [34], ARCO [35], Relief [36] and mRMR [37]. The SVM-REF relies on constructing feature ranking coefficients based on the weight vector generated by SVM during training. Under this approach, a feature with the smallest ranking coefficient in each iteration is removed, until finally obtaining a descending ranking of all feature attributes. Area under the Receiver Operating Characteristic Curve (AUC) has been commonly used by the machine learning community in feature selection. The Relief algorithm is a feature weighting algorithm, which assigns different weights to features according to the correlation of each feature and category, and features whose weight are less than a certain threshold are removed. The mRMR algorithm was used to ensure the maximum correlation while removing redundant features, which is equivalent to obtaining a set of “purest” feature subsets. This is particularly useful when the features are very different. For implementation of classification models using ML algorithms and performance measurements, we used the Waikato Environment for Knowledge Analysis (WEKA) [38], an open source implemented in the Java-based framework.

## 3. Results

### 3.1. Result of Differential Expression and Feature Selection

The objective of this investigation was to identify a set of significantly (*p* < 0.05) differentially expressed genes that could distinguish TNBC from non-TNBC, and could be used as features for developing algorithms for classification of the two types of BC. We hypothesized that genomic alterations in women diagnosed with TNBC and those diagnosed with non-TNBC could lead to measurable changes distinguishing the two types of BC. To address this hypothesis, we performed whole transcriptome analysis comparing gene expression levels between TNBC and non-TNBC. The genes were ranked based on estimates of *p*-values and logFC. Only significantly (*p* < 0.05) differentially expressed genes with a high logFC identified after correcting for multiple hypothesis testing were selected and used as features in model development and validation. Note that all the estimates of the *p*-values were adjusted for multiple hypothesis testing using the false discovery rate procedure [30]. The analysis produced a signature of 5502 significantly (*p* < 0.05, |logFC| > 1) differentially expressed genes distinguishing patients with TNBC from non-TNBC. A summary of the results showing the top 30 most highly significantly differentially expressed genes along with estimates of *p*-value and logFC are presented in Table 1. A complete list of all the 5502 significantly (*p* < 0.05, |logFC| > 1) differentially expressed genes is presented in Supplementary Table S1. 

### 3.2. Result of Classification

The objective of this investigation was to develop a classification algorithm based on ML that could accurately identify genes distinguishing truly TNBC from non-TNBC. The rationale was that molecular based classification using ML algorithms could provide a framework to accurately identify women at high risk of developing TNBC that could be prioritized for specialized treatment. To address this need, we evaluated the performance of four classification algorithms using the 5502 significantly differentially expressed genes identified from differential gene expression analysis using different threshold levels (*p*-values). The evaluated classifiers included the kNNs, NGB, DT and SVM. Each of these classifiers was modeled 10 times. Each algorithm was evaluated for accuracy, sensitivity/recall and specificty, computed as averages of the number of times each was modeled. The results showing accuracy, recall and specificity for the four classification algorithms computed as averages are shown in Table 2.

Among the four classification algorithms evaluated, SVM had the best performance with an accuracy of 90%, a recall of 87% and a specificty of 90%, followed by KNN, with an accuracy of 87%, a recall of 76 and specificty of 88%. Although NGB and DT were relatively accurate, they performed badly on recall. The variability in the evaluation parameters can be partially explained by the large numbers of features used and the unbalanced study design.

**Figure 2:**
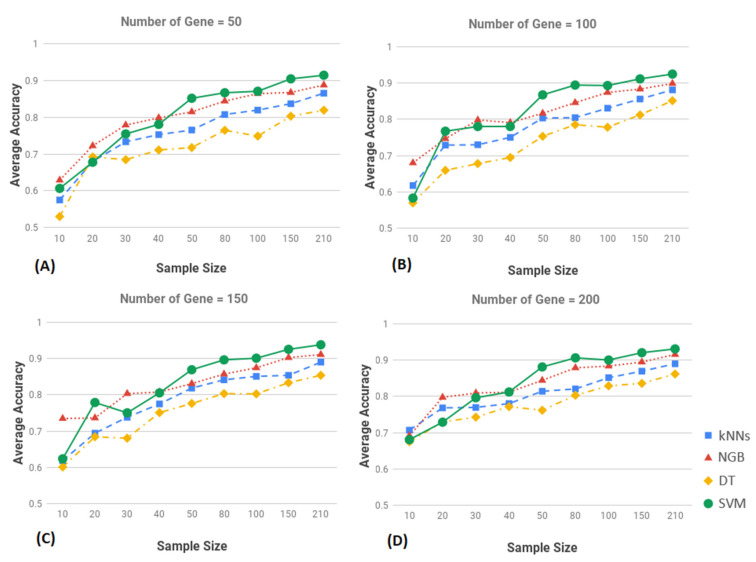
Classification average accuracy of machine learning (ML) methods of different training sample and top k-gene markers, k = 50 (**A**), k = 100 (**B**), k = 150 (**C**), and k = 200 (**D**), where k is the number of the top most highly significant genes used for various algorithms in each subfigure, on the training and the test sets of breast cancer (BC). For each panel, the *x*-axis is the sample size used for training, and the *y*-axis represents the classification accuracy. The blue, red, yellow and green lines represent the K-nearest neighbors (kNNs), Naïve Bayes (NGB), Decision tree (DT) and Support Vector Machines (SVM) method, respectively.

As noted above, the large number of features (5502 genes) can affect the performance of the classification algorithms. Therefore, to determine the optimal performance of each algorithm, we performed addition tests on the algorithms using smaller numbers of genes selected using different threshold levels. Under this approach the 5502 genes were ranked on FDR adjusted *p*-values. We selected the top 200, 150, 100 and 50 genes for use in the performance evaluation of each model using the same parameters as above, accuracy, recall and specificity. For each set of genes, we tested the performance of all four algorithms. The results of this investigation are presented in Figure 2 with plots showing the performance of each model under a specified number of genes plotted as a function of sample size. In the figure the *x*-axis accuracy shows the sample size and *y*-axis shows the accuracy.

The results show that the performance of each algorithm as function of sample size was relatively consistent. The performance of all classification algorithms increased with increasing sample size (Figure 2). No single classification technique proved to be significantly superior to all others in the experiments we performed (Figure 2). This can partially be explained by the small samples used in the investigation and the unbalanced design of the study project. In general, the plot showed that the SVM algorithm was better than the other three algorithms at higher sample sizes, i.e., greater than 50 (Figure 2). The DT algorithms performed worse than the others.

### 3.3. Performance Evalaution of SVM

Following evaluation of all the four algorithms and the discovery that SVM had the best performance, we decided to test this algorithm using different numbers to determine its robustness. We evaluated this algorithm using varying numbers of significant genes as determine by *p*-value and FDR. That is from 1 to 5502 genes. The tests were performed using the same parameters as those above using these smaller feature sets.

**Figure 3:**
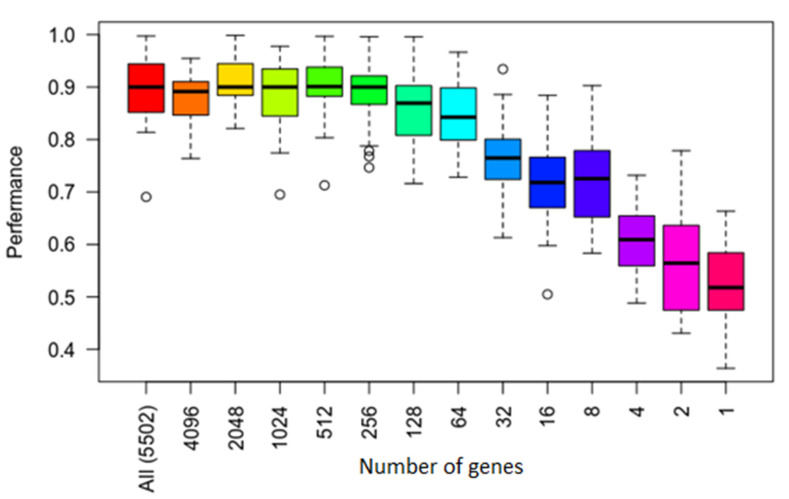
Average accuracy at varying levels of training sample and gene sizes of Support Vector Machines (SVM) method. The *x*-axis represents the top number of genes, and the *y*-axis represents the average accuracy. The top and bottom of the box are the 75th and 25th percentiles, respectively. The top and bottom bar are the maximum and minimum value. The circles are the outliers.

Figure 3 shows results of performance for each number of genes and for overall significant genes. The top and bottom of the box are the 75th and 25th percentiles, respectively. The top and bottom bar are the maximum and minimum value. The circles are the outliers. Figure 3 shows that performance variance was larger when the number of genes was less.

The results showing details of model performance using the training and test sets are shown in Table 3 which displays the most significant results from these experiments. As shown in Figure 3 and Table 3, the best classification performance was achieved using the top 256 genes as features. In general, the smaller sets of genes achieved slightly better scores compared to using all features/genes, though the improvement was not highly significant.

### 3.4. Comparative Evaluation and Validation of SVM Results

To further validate the developed algorithms, we compared the classification results from this investigation with classic feature selection methods such as SVM-RFE (SVM-Recursive Feature Elimination) [34], ARCO ((Area Under the Curve (AUC) and Rank Correlation coefficient Optimization) [35], Relief [36] and mRMR (minimal redundancy-maximal-relevance) [37] using our data. The mRMR method recorded the highest classification when the number of features/genes was 32, which recorded an accuracy of 83%. The ARCO method achieved the highest classification accuracy (82%) with 64 feature genes. The SVM-RFE method produced the highest classification accuracy (73%) with 128 feature genes, whereas the Relief method recorded the highest classification accuracy (70) with 16 feature genes. As evidenced, the classification accuracy of the above methods was lower than the classification of BC into TNBC and non-TNBC models developed and implemented in this investigation.

## 4. Discussion

We evaluated the performance of four ML-based classification algorithms: kNNs, NGB, DT and SVM for classification of breast cancer into TNBC and non-TNBC using gene expression data. The investigation revealed that ML algorithms could classify BC into TNBC and non-TNBC. SVM algorithm was the most accurate among the four algorithms. This is consistent with previous reoprts [39]. Nindrea et al. compared SVM to artificial neural network (ANN), decision tree (DT), Naïve Bayes (NB) and K-Nearest Neighbor (KNN) in a meta-analysis of classification algorithms in BC and found that SVM was superior to the other three algorithms [39]. BC classification using imaging data has also been reported [40].

The main difference and novel aspect of our investigation is that it is the first study to report application of ML to classification of BC into TNBC and non-TNBC using RNA-seq data. The clinical significance of this investigation is that ML algorithms could be used not only to improve diagnostic accuracy, but also for identifying women at high risk of developing TNBC which could be prioritized for treatment.

As noted earlier in this report, breast cancer is a highly heterogeneous disease. Thus, one of the major challenges is building accurate and computationally efficient algorithms for classifying patients to guide therapeutic decision making at the point of care. Our investigation shows that among ML-based classification algorithms, SVM out performed the other algorithms and provides the best framewrok for BC classification. This is consistent with previous reports [41,42,43,44]. The clinical significance is that, in addition to classification of BC into TNBC and non-TNBC as demonstrated in this investigation, SVM could also be used for efficient risk, diagnosis and outcome predictions where it has been reported to be superior to other algorithms [41,42,43,44]. Althouh we did not investigate use of ML and in particular SVM algorithm for risk, diagnosis and outcome prediction in this investigation, several studies have reported such application in BC and have also shown its superiority to other algorithms [41,42,43,44], which is consistent with our investigation.

Traditional classification of TNBC and non-TNBC involves use of immunohstochemical staining conducted by hispothologists. In addition, imaging has been used extensively in BC classification [19,40] and radiomics is increasingly being used as a diagnostic tool [20,21]. While there is no doubt that BC clasification using histopathology, imaging and radiomics has been relatively effective, ML algorithms proposed in this investigation provides a novel framework for accurate classification of BC tumors into TNBC and non-TNBC and could complement imaging modalities. More importantly, ML algorithms could help reduce the possible human errors that can occurr because of fatigued or inexperienced experts when medical data is to be examined in shorter time and in more detail. Moreover, given the aggressivenees and lethality of TNBC, accurate identifification of patients with this lethal disease in the early stages may lead to early interventions and improved outcomes.

Our investigation revealed that ML algorithms offer the potential for classifying BC into TNBC and non-TNBC. However, limitations of the study must be acknowledged. First the data size was relatively small and the design was unbalanced with TNBC samples being significantly fewer than non-TNBC. This has the practical consequence of reducing the statistical power of models and also introducing sampling errors in feature selections from differentiall expression analysis. Second, although our ML evaluated and compared the performance of four algorithms, there are many other algorithms that we did not evaluate. However, not withstanding this weakness, evaluation of other algorithms has shown that SVM is superior in BC classification [41,42,43,44]. Lastly, but not least, both TNBC and non-TNBC consist of multiple subtypes of BC and the proposed ML algorithms did not address that problem, as such an undertaking was beyond the scope of this investigation given the small samples sizes and lack of information for ascertaining subtypes.

## 5. Conclusions

The investigation revealed that ML algorithms can accurately classify BC into the two primary types, TNBC and non-TNBC. The investigation confirmed that the SVM algorithm is able to calculately classify BC into TNBC and non-TNBC more accurately, and with more sensitivity, specificity and lower misclassification errors than other ML algorithms. Further research is recommended to investigate the power of ML algorithms in classifications of subtypes of TNBC and non-TNBC, to identify the best classification features and to integrate radiomics with genomics data. These are subjects of our future investigations.

## 6. Patents

No patents resulted from the work reported in this manuscript.

## Figures and Tables

**Table 1:** Top 30 significantly differentially expressed genes distinguishing TNBC from non-TNBC.

Gene Name	Chromosome	Log2 Fold Change (logFC)	Adjust *p*-Value
*ESR1*	6q25.1-q25.2	−8.966061547	1.02 × 10^−35^
*MLPH*	2q37.3	−6.231155611	1.02 × 10^−35^
*FSIP1*	15q14	−6.785688629	2.04 × 10^−35^
*C5AR2*	19q13.32	−4.919151624	3.08 × 10^−35^
*GATA3*	10p14	−5.490221514	4.68 × 10^−35^
*TBC1D9*	4q31.21	−4.720190121	8.82 × 10^−35^
*CT62*	15q23	−8.112412605	9.86 × 10^−35^
*TFF1*	21q22.3	−13.06903719	2.16 × 10^−34^
*PRR15*	7p14.3	−6.25260355	2.16 × 10^−34^
*CA12*	15q22.2	−6.168504259	2.16 × 10^−34^
*AGR3*	7p21.1	−11.46873847	2.38 × 10^−34^
*SRARP*	1p36.13	−12.26807072	7.31 × 10^−34^
*AGR2*	7p21.1	−8.8234708	1.32 × 10^−33^
*BCAS1*	20q13.2	−6.465140066	1.34 × 10^−33^
*LINC00504*	4p15.33	−7.846987181	2.13 × 10^−33^
*THSD4*	15q23	−5.0752667	2.13 × 10^−33^
*CCDC170*	6q25.1	−5.019657927	2.13 × 10^−33^
*RHOB*	2p24.1	−2.828470443	2.13 × 10^−33^
*FOXA1*	14q21.1	−8.268856317	2.78 × 10^−33^
*ZNF552*	19q13.43	−3.813954916	2.78 × 10^−33^
*SLC16A6*	17q24.2	−4.45954505	2.99 × 10^−33^
*CFAP61*	20p11.23	−3.680660547	4.88 × 10^−33^
*GTF2IP7*	7q11.23	−6.49829058	4.98 × 10^−33^
*NEK5*	13q14.3	−3.666310207	5.90 × 10^−33^
*TTC6*	14q21.1	−7.69269993	1.00 × 10^−32^
*HID1*	17q25.1	−3.069655358	1.00 × 10^−32^
*ANXA9*	1q21.3	−3.748683928	1.45 × 10^−32^
*AK8*	9q34.13	−3.134793023	1.45 × 10^−32^
*FAM198B-AS1*	4q32.1	−4.757293943	1.63 × 10^−32^
*NAT1*	8p22	−6.278947772	3.24 × 10^−32^

**Table 2:** Performance of classification model for 5502 signature genes.

	Accuracy	Recall	Specificity
K-nearest neighbor (kNN)	87%	76%	88%
Naïve Bayes(NGB)	85%	68%	87%
Decision trees (DT)	87%	54%	91%
Support Vector Machines (SVM)	90%	87%	90%

**Table 3:** SVM classifier trained on SVM genes obtained with the DE method.

Number of Genes	Training Set					Test Set				
	Accuracy	Precision	Recall	Specify	F1 Score	Accuracy	Precision	Recall	Specify	F1 Score
All (5502)	0.90	0.51	0.87	0.90	0.65	0.82	0.33	0.67	0.80	0.44
4096	0.90	0.52	0.88	0.91	0.65	0.85	0.37	0.58	0.71	0.45
2048	0.92	0.56	0.86	0.92	0.68	0.84	0.38	0.75	0.83	0.50
1024	0.91	0.53	0.87	0.91	0.66	0.86	0.41	0.75	0.81	0.53
512	0.90	0.51	0.88	0.90	0.65	0.83	0.33	0.58	0.74	0.42
256	0.91	0.53	0.89	0.91	0.67	0.85	0.38	0.67	0.76	0.48
128	0.89	0.49	0.87	0.90	0.63	0.82	0.35	0.75	0.85	0.47
64	0.87	0.44	0.78	0.88	0.56	0.76	0.26	0.67	0.85	0.37
32	0.78	0.27	0.64	0.80	0.38	0.71	0.19	0.50	0.81	0.27
16	0.74	0.22	0.63	0.75	0.33	0.69	0.21	0.67	0.89	0.31

Accuracy = (TP + TN)/(TP + FP + FN + TN). Precision = TP/(TP + FP). Recall = TP/(TP + FN). F1 Score = 2 * (Recall * Precision)/(Recall + Precision). Specificity = TN/(TN + FP).

## Data Availability

The data that support the findings of this study are provided in supplementary tables as documented below, and original data sets are also made available in the TCGA (https://www.cancer.gov/about-nci/organization/ccg/research/structural-genomics/tcga) and are downloadable via the Genomics Data Commons https://gdc.cancer.gov/.

## Supplementary Materials

The following are available online at https://www.mdpi.com/2075-4426/11/2/61/s1. Supplementary Table S1 complete list of significantly differentially expressed genes distinguishing TNBC from non-TNBC.

## References

[1] Siegel R.L., Miller K.D., Jemal A. (2019). Cancer Statistics, 2019. CA Cancer J. Clin..

[2] American Cancer Society (2019). Cancer Facts and Figures Report 2019.

[3] Dietze E.C., Sistrunk C., Miranda-Carboni G., O’Regan R., Seewaldt V.L. (2015). Triple-negative breast cancer in African-American women: Disparities versus biology. Nat. Rev. Cancer.

[4] Perou C.M. (2010). Molecular Stratification of Triple-Negative Breast Cancers. Oncologist.

[5] Xu H., Eirew P., Mullaly S.C., Aparicio S. (2014). The omics of triple-negative breast cancers. Clin. Chem..

[6] Homero G., Maximiliano R.G., Jane R., Duarte C. (2018). Survival Study of Triple-Negative and Non-Triple-Negative Breast Cancer in a Brazilian Cohort. Clin. Med. Insights Oncol..

[7] Joyce D.P., Murphy D., Lowery A.J., Curran C., Barry K., Malone C., McLaughlin R., Kerin M.J. (2017). Prospective comparison of outcome after treatment for triple-negative and non-triple-negative breast cancer. Surgeon.

[8] Li X., Yang J., Peng L., Sahin A.A., Huo L., Ward K.C., O’Regan R., Torres M.A., Meisel J.L. (2017). Triple-negative breast cancer has worse overall survival and cause-specific survival than non-triple-negative breast cancer. Breast Cancer Res. Treat..

[9] Pan X.B., Qu S., Jiang Y.M., Zhu X.D. (2015). Triple Negative Breast Cancer versus Non-Triple Negative Breast Cancer Treated with Breast Conservation Surgery Followed by Radiotherapy: A Systematic Review and Meta-Analysis. Breast Care.

[10] Ye J., Xia X., Dong W., Hao H., Meng L., Yang Y., Wang R., Lyu Y., Liu Y. (2016). Cellular uptake mechanism and comparative evaluation of antineoplastic e_ects of paclitaxel-cholesterol lipid emulsion on triple-negative and non-triple-negative breast cancer cell lines. Int. J. Nanomed..

[11] Qiu J., Xue X., Hu C., Xu H., Kou D., Li R., Li M. (2016). Comparison of Clinicopathological Features and Prognosis in Triple-Negative and Non-Triple Negative Breast Cancer. J. Cancer.

[12] Podo F., Santoro F., di Leo G., Manoukian S., de Giacomi C., Corcione S., Cortesi L., Carbonaro L.A., Trimboli R.M., Cilotti A. (2016). Triple-Negative versus Non-Triple-Negative Breast Cancers in High-Risk Women: Phenotype Features and Survival from the HIBCRIT-1 MRI-Including Screening Study. Clin. Cancer Res..

[13] Nabi M.G., Ahangar A., Wahid M.A., Kuchay S. (2015). Clinicopathological comparison of triple negative breast cancers with non-triple negative breast cancers in a hospital in North India. Niger. J. Clin. Pract..

[14] Koshy N., Quispe D., Shi R., Mansour R., Burton G.V. (2010). Cisplatin-gemcitabine therapy in metastatic breast cancer: Improved outcome in triple negative breast cancer patients compared to non-triple negative patients. Breast.

[15] Milica N., Ana D. (2019). Mechanisms of Chemotherapy Resistance in Triple-Negative Breast Cancer-How We Can Rise to the Challenge. Cells.

[16] Giuseppe V., Leen S., de Snoo F.A. (2016). Discordant assessment of tumor biomarkers by histopathological and molecular assays in the EORTC randomized controlled 10041/BIG 03-04 MINDACT trial breast cancer: Intratumoral heterogeneity and DCIS or normal tissue components are unlikely to be the cause of discordance. Breast Cancer Res. Treat..

[17] Viale G., de Snoo F.A., Slaets L., Bogaerts J. (2018). Immunohistochemical versus molecular (BluePrint and MammaPrint) subtyping of breast carcinoma. Outcome results from the EORTC 10041/BIG 3-04 MINDACT trial. Breast Cancer Res. Treat..

[18] Michael U., Bernd G., Nadia H. (2013). Gallen international breast cancer conference 2013: Primary therapy of early breast cancer evidence, controversies, consensus—Opinion of a german team of experts (zurich 2013). Breast Care.

[19] Annarita F., Teresa M.B., Liliana L. (2019). Ensemble Discrete Wavelet Transform and Gray-Level Co-Occurrence Matrix for Microcalcification Cluster Classification in Digital Mammography. Appl. Sci..

[20] Liliana L., Annarita F., Teresa M., Basile A. (2019). Radiomics Analysis on Contrast-Enhanced Spectral Mammography Images for Breast Cancer Diagnosis: A Pilot Study. Entropy.

[21] Allegra C., Andrea D., Iole I. (2020). Radiomics in breast cancer classification and prediction. Seminars Cancer Biology.

[22] Mitra M., Mohadeseh M., Mahdieh M., Amin B. (2016). Machine learning models in breast cancer survival prediction. Technol. Health Care.

[23] Tong W., Laith R.S., Jiawei T., Theodore W.C., Chandra M.S. (2019). Machine learning for diagnostic ultrasound of triple-negative breast cancer. Breast Cancer Res. Treat..

[24] Riku T., Dmitrii B., Mikael L. (2019). Breast cancer outcome prediction with tumour tissue images and machine learning. Breast Cancer Res. Treat.

[25] Weinstein J.N., Collisson E.A., The Cancer Genome Atlas Research Network (2013). The Cancer Genome Atlas Pan-Cancer analysis project. Nat. Genet..

[26] National Cancer Institute The Genomics Data Commons. https://gdc.cancer.gov/.

[27] Ritchie M.E., Phipson B., Wu D. (2015). limma powers differential expression analyses for RNA-sequencing and microarray studies. Nucleic Acids Res..

[28] Kas K., Schoenmakers E.F., Van de Ven W.J. (1995). Physical map location of the human carboxypeptidase M gene (CPM) distal to D12S375 and proximal to D12S8 at chromosome 12q15. Genomics.

[29] Mihaly V., Peter T. (2015). The Protein Ensemble Database. Adv. Exp. Med. Biol..

[30] Benjamini Y., Yosef H. (1995). Controlling the false discovery rate: A practical and powerful approach to multiple testing. J. R. Stat Soc..

[31] Shawe-Taylor J., Nello C. (2004). Kernel Methods for Pattern Analysis.

[32] Bernhard S., Smola A.J. (2002). Learning with Kernels.

[33] Powers D.M.W. (2011). Evaluation: From Precision, Recall and F-Measure to ROC, Informedness, Markedness & Correlation. J. Mach. Learn. Technol..

[34] Huang M.L., Hung Y.H., Lee W.M., Li R.K., Jiang B.R. (2014). SVM-RFE based feature selection and Taguchi parameters optimization for multiclass SVM classifier. Sci. World J..

[35] Piñero P., Arco L., García M.M., Caballero Y., Yzquierdo R., Morales A., Sanfeliu A., Ruiz-Shulcloper J. (2003). Two New Metrics for Feature Selection in Pattern Recognition. Progress in Pattern Recognition, Speech and Image Analysis. CIARP 2003. Lecture Notes in Computer Science.

[36] Kira K., Rendell L. The Feature Selection Problem: Traditional Methods and a New Algorithm. Proceedings of the AAAI-92 Proceedings.

[37] Auffarth B., Lopez M., Cerquides J. Comparison of redundancy and relevance measures for feature selection in tissue classification of CT images. Proceedings of the Industrial Conference on Data Mining.

[38] Tony C.S., Eibe F. (2016). Introducing Machine Learning Concepts with WEKA. Methods Mol. Biol..

[39] Ricvan D.N., Teguh A., Lutfan L., Iwan D. (2018). Diagnostic Accuracy of Different Machine Learning Algorithms for Breast Cancer Risk Calculation: A Meta-Analysis. Asian Pac. J. Cancer Prev..

[40] La Forgia D. (2020). Radiomic Analysis in Contrast-Enhanced Spectral Mammography for Predicting Breast Cancer Histological Outcome. Diagnostics.

[41] Asri H., Mousannif H., Al Moatassime H., Noel T. (2016). Using machine learning algorithms for breast cancer risk prediction and diagnosis. Procedia Comput. Sci..

[42] Polat K., Gunes S. (2007). Breast cancer diagnosis using least square support vector machine. Digit. Signal Process.

[43] Akay M.F. (2006). Support vector machines combined with feature selection for breast cancer diagnosis. Expert Syst. Appl..

[44] Heidari M., Khuzani A.Z., Hollingsworth A.B. (2018). Prediction of breast cancer risk using a machine learning approach embedded with a locality preserving projection algorithm. Phys. Med. Biol..

